# Durable remission in a patient with *ERBB2*-amplified recurrent mucinous ovarian carcinoma treated with Trastuzumab-Carboplatin-Paclitaxel

**DOI:** 10.1016/j.gore.2023.101237

**Published:** 2023-07-07

**Authors:** Alexander J. Neil, Michael G. Muto, David L. Kolin, Panagiotis A. Konstantinopoulos

**Affiliations:** a Department of Pathology, Brigham and Women’s Hospital, Boston, MA, USA; b Division of Gynecologic Oncology and Reproductive Biology, Brigham and Women's Hospital, Boston, MA, USA; c Department of Medical Oncology, Dana Farber Cancer Institute, Boston, MA, USA

**Keywords:** Mucinous ovarian cancer, HER2, ERBB2, Trastuzumab, Carboplatin/Paclitaxel chemotherapy, Expansile growth

## Abstract

Patients with advanced stage or recurrent mucinous ovarian carcinoma exhibit poor response to standard platinum- and taxane-based chemotherapy and poor prognosis. We report a 29-year-old patient with recurrent *ERBB2*-amplified mucinous ovarian carcinoma (with expansile growth pattern at initial diagnosis and previously treated with adjuvant capecitabine/oxaliplatin) who underwent optimal secondary cytoreduction followed by 6 cycles of carboplatin/paclitaxel/trastuzumab and 1-year maintenance trastuzumab. This patient remains without radiologic or biochemical evidence of disease for more than 3 years after secondary cytoreduction. This case supports routine assessment of HER2 status in patients with advanced or recurrent mucinous ovarian carcinoma and highlights the potential of HER2-targeted therapy with trastuzumab in combination with standard carboplatin and paclitaxel in this disease. This case also raises the possibility that expansile mucinous ovarian carcinomas with *ERBB2* amplification and p53 mutant immunohistochemical staining pattern (as this patient had) may be associated with a more aggressive behavior and higher risk of relapse.

## Highlights

• Patients with advanced or recurrent mucinous ovarian carcinoma exhibit poor response to chemotherapy and poor prognosis.
• We report marked response to carboplatin/paclitaxel/trastuzumab in recurrent *ERBB2*-amplified mucinous ovarian carcinoma.
• This case supports routine assessment of HER2 status in patients with advanced or recurrent mucinous ovarian carcinoma.

## 1 Introduction

Primary mucinous tumors of the ovary comprise a spectrum of histologically distinct entities, ranging from benign cystadenomas to borderline tumors to carcinoma, with differing risk of recurrence and disease-associated death (Morice et al., 2019). A spectrum of histology, from benign cystadenoma to carcinoma, may be present in a single tumor, and all mucinous ovarian neoplasms share critical oncogenic driver mutations in *KRAS* and *CDKN2A* (Hunter et al., 2012, Mackenzie et al., 2015), suggesting that carcinomas represent progression from borderline and adenomatous precursors. The cell of origin in mucinous ovarian tumors remains elusive, however clinical and genomic evidence support these tumors as primary ovarian neoplasms despite their histologic overlap with mucinous tumors of other sites (Cheasley et al., 2019). In cases of stage I expansile mucinous carcinoma, the prognosis is usually excellent. However, high stage (extra-ovarian disease) is often associated with chemoresistance and a poor prognosis (Morice et al., 2019).

Amplification of the *ERBB2* gene on chromosome 17, which encodes the human epidermal growth factor receptor 2 protein (HER2), is a well-characterized targetable finding in tumors of the upper gastrointestinal tract, breast, and endometrium. Trastuzumab is a humanized monoclonal antibody against HER2 that was first approved by the Food and Drug Administration in HER2 overexpressing metastatic breast cancer either as a single agent in women who have received one or more chemotherapy regimens or in combination with paclitaxel for first-line treatment. It is also FDA approved for HER2 overexpressing gastric cancers in combination with cisplatin and capecitabine or 5-fluorouracil. Pan-cancer analyses show that this oncogenic driver event is found in up to 3.4% of all human solid cancers, which has led to increased interest in expanding the usage of trastuzumab beyond these tumor types (Daemen and Manning, 2018). *ERBB2* amplification is identified in up to 6.6% of all ovarian epithelial carcinomas (Tuefferd et al., 2007) and is more commonly seen (18 to 26%) in mucinous ovarian carcinomas (Mackenzie et al., 2015, Cheasley et al., 2019, Anglesio et al., 2013, Gorringe et al., 2020, McAlpine et al., 2009). *ERBB2* amplification is found with greater frequency in mucinous ovarian carcinoma than in mucinous borderline ovarian tumors, suggesting it is a key genomic event in tumor progression (Mackenzie et al., 2015, Cheasley et al., 2019).

HER2-targeted therapies have been evaluated in the treatment of ovarian carcinomas previously with only modest activity (Bookman et al., 2003, Guastalla et al., 2007, Kurzeder et al., 2016). However, these studies included primarily patients with high-grade serous carcinoma. No randomized controlled trials have focused on the therapeutic benefit of HER2-directed therapy specifically in mucinous ovarian carcinoma, which genetically shares greater overlap with mucinous carcinomas of the pancreaticobiliary tract and colon than other epithelial ovarian carcinomas (Cheasley et al., 2019). Notably, women with stage III or IV or recurrent mucinous ovarian carcinoma have significantly lower overall survival compared to women with non-mucinous ovarian epithelial carcinoma, suggesting novel therapeutic strategies are needed for these patients to improve outcomes (Morice et al., 2019).

*TP53* mutations occur in up to 64% of mucinous ovarian carcinomas based on sequencing data and immunohistochemical analyses (Cheasley et al., 2019, Kang et al., 2021). p53 mutant status as determined by immunohistochemistry is associated with higher risk of recurrence and disease specific death in mucinous borderline tumors but is not associated with worse outcomes in carcinomas (Kang et al., 2021). *TP53* mutations and *ERBB2* amplification frequently co-occur in mucinous carcinomas (Cheasley et al., 2019).

## 2 Case presentation

A 29-year-old woman with no significant past medical history presented with right upper quadrant pain, abdominal distension, and nausea. A pelvic ultrasound and subsequent CT of the abdomen and pelvis demonstrated a 21 cm right ovarian mass with free fluid in the lower pelvis and no lymphadenopathy. Exploratory laparotomy and cystectomy were performed with 5L of fluid drained from the cyst. On gross pathologic examination, the cystectomy specimen was disrupted, was 7.8 cm in greatest dimension and was without grossly identifiable excrescences. Histologic examination revealed a mucinous borderline tumor without ovarian surface involvement.

**Fig. 1:**
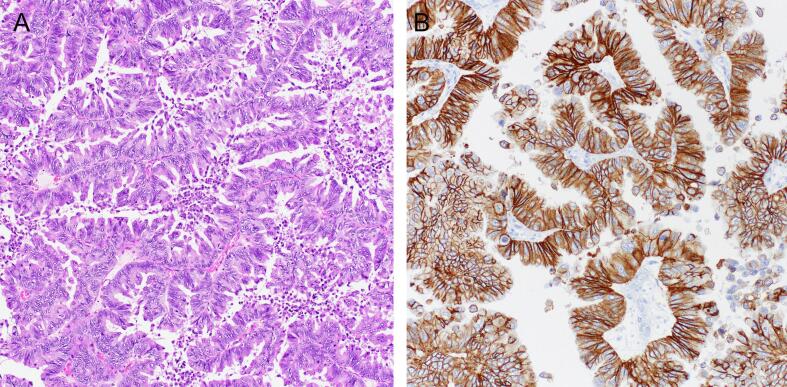
(A) Mucinous ovarian carcinoma with expansile growth and papillary architecture (200X). (B) HER2 immunohistochemistry showing strong and diffuse basolateral staining (3+, positive, 200X).

Laparoscopic right salpingo-oophorectomy, pelvic washings and omental biopsy were then performed with free fluid noted in the pelvis but no gross evidence of disseminated disease. Gross examination of the salpingo-oophorectomy specimen demonstrated a disrupted 10.0 cm cystic ovary with bosselated cyst lining. Histologic examination revealed an expansile mucinous adenocarcinoma with papillary and confluent glandular architecture (Fig. 1A). There was no evidence of infiltrative invasion. The ovarian surface was involved, and the pelvic washings were positive for mucinous adenocarcinoma. The omental biopsy was negative for tumor. p53 immunohistochemistry demonstrated mutant null staining in tumor cells.

Molecular profiling of the ovarian tumor was performed using a targeted 447 gene next generation sequencing panel, as previously described (Garcia et al., 2017). Sequencing revealed a tumor mutational burden of 3.0 mutations/megabase, a pathogenic *KRAS* p.G12D variant, amplification of *ERBB2*, and copy number loss of *CDKN2A* and *RB1*. Microsatellite instability was not detected. A pathogenic *MUTYH* p.G396D variant was identified at 54% variant allele fraction, which was subsequently confirmed to be a germline alteration via the CancerNext-*Expanded* panel (Ambry Genetics, Aliso Viejo, CA). Her family history included a father diagnosed with a kidney tumor at age 63, a maternal grandmother diagnosed with breast cancer at age 68, a paternal grandfather diagnosed with prostate cancer and a paternal grandmother diagnosed with a brain tumor.

Post-surgical CA125 was 97 U/mL (normal range 6–38). She received 3 cycles of adjuvant capecitabine/oxaliplatin with nadir CA125 of 18 U/mL. Follow up CT abdomen pelvis 5 months after completion of chemotherapy showed increase in size of the left ovary with pelvic peritoneal nodularity and free fluid, concerning for metastatic recurrence. CA125 at that time was 249 U/mL. Exploratory laparotomy revealed free fluid in the pelvis and mucinous implants diffusely involving the left round ligament, anterior cul-de-sac, posterior wall of the uterus and both uterosacral ligaments. Biopsies were obtained and showed metastatic mucinous adenocarcinoma involving the left ovary, rectosigmoid colon and uterus. Immunohistochemistry demonstrated diffuse basolateral positivity for HER2 (Fig. 1B) and intact staining for mismatch repair proteins. Peritoneal fluid was positive for mucinous adenocarcinoma. Repeat panel NGS testing confirmed the presence of *ERBB2* amplification (estimated copy number 41) and *KRAS* p.G12D with a more complex array of copy number alterations compared to the prior testing including chromosomal level loss of chromosome 9, and arm level loss of chromosome 17p including the *TP53* locus.

Optimal secondary cytoreductive surgery including total abdominal hysterectomy and left salpingo-oophorectomy was performed. Post-operative CA125 was 23 U/mL. She received 6 cycles of carboplatin, paclitaxel and trastuzumab followed by 1 year of maintenance trastuzumab. CA125 was stable at 12–14 U/mL following initial chemotherapy. She remains without radiologic or biochemical evidence of disease more than 3 years after secondary cytoreduction (2 years after completion of trastuzumab maintenance). This patient consented to allow discussion of her case and inclusion of pertinent images in this report.

## 3 Discussion

We describe durable remission in a 29-year-old woman with recurrent metastatic *ERBB2*-amplified mucinous ovarian adenocarcinoma following optimal secondary cytoreduction and carboplatin/paclitaxel/trastuzumab followed by one year of maintenance trastuzumab. This study highlights the therapeutic potential of HER2-directed therapies specifically in mucinous ovarian carcinomas. Trastuzumab monotherapy or combinations of trastuzumab with carboplatin or weekly paclitaxel or lapatinib as single agents have been previously reported in patients with recurrent *ERBB2*-amplified or overexpressing recurrent mucinous ovarian carcinomas but responses in all these cases were relatively short in duration (McAlpine et al., 2009, Jain et al., 2012). To our knowledge, this is the first report of the carboplatin/paclitaxel/trastuzumab regimen followed by one year of maintenance trastuzumab in a patient with *ERBB2*-amplified mucinous ovarian carcinoma and the first report of a durable remission in this setting. Given the extensive experience with addition of trastuzumab to standard chemotherapy in breast and gastric cancers, we reasoned that addition of trastuzumab to standard carboplatin/paclitaxel chemotherapy would be a reasonable approach for this patient who was previously treated with capecitabine/oxaliplatin. Maintenance trastuzumab for 1 year after 6 cycles of carboplatin/paclitaxel/trastuzumab was thus recommended in an effort to further reduce the risk of recurrence following the breast cancer treatment paradigm. Given the relatively high frequency of *ERBB2* amplification in mucinous carcinomas (18–26%) (Cheasley et al., 2019, McAlpine et al., 2009), the potential benefit of this targeted approach may have clinical relevance to a substantial portion of patients with high stage disease as well as those with stage I disease and other risk factors, such as infiltrative invasion or surface involvement.

At initial resection, this patient had an expansile mucinous carcinoma, with surface ovarian involvement and positive pelvic wash cytology (FIGO stage IC3). National Comprehensive Cancer Network (NCCN) guidelines (which do not incorporate the distinction between expansile vs infiltrative pattern of invasion) suggest monitoring or chemotherapy in this setting while the ESMO-ESGO guidelines (which incorporate the distinction between expansile vs infiltrative pattern of invasion) suggest that adjuvant chemotherapy is optional for stage IC3, expansile mucinous ovarian carcinomas. Determining when to opt for systemic therapy for these patients can be driven by multiple factors, in particular the histologic pattern of invasion (expansive vs infiltrative). Prior studies suggest a recurrence rate of 3–4% in patients with stage I disease and expansile histology as opposed to 15–30% with stage I disease and infiltrative histology (Morice et al., 2019). In this instance, the presence of a p53 mutant immunohistochemical staining pattern suggested that her tumor may behave more aggressively despite the expansile growth pattern and drove the decision to treat with 3 cycles of adjuvant capecitabine/oxaliplatin. It is also possible that *ERBB2* amplification may be associated with a more aggressive behavior in the setting of an expansile growth pattern; further studies are needed to answer this question.

One of the few mucinous ovarian carcinoma-specific randomized controlled trials compared the efficacy of capecitabine/oxaliplatin versus paclitaxel/carboplatin plus or minus bevacizumab (GOG 241 trial). Preliminary results based on a limited number of patients showed no significant differences between objective response rate in the experimental arms and the trial was ultimately halted due to poor accrual (Gore et al., 2019). GOG 241 highlights the challenges of performing randomized clinical trials in patients with rare histologies (like mucinous ovarian carcinomas) as well as the potential utility of isolated case reports in informing clinical management of patients with rare tumors (in this case *ERBB2*-amplified mucinous ovarian carcinomas).

Of note, our patient possesses a germline *MUTYH* p.G396D germline alteration. *MUTYH* encodes a DNA glycosylase involved in the repair of mismatched adenines incorporated opposite damaged 8-hydroxyguanine during DNA replication. This variant is annotated as pathogenic in ClinVar (VCV000005294.90) and is associated with familial adenomatous polyposis 2, an autosomal recessive cancer predisposition syndrome characterized by multiple colorectal adenomas and an increased risk of colorectal cancer, urinary bladder cancer and ovarian cancer (Win et al., 2016) (OMIM #608456). Monoallelic mutation carrier status, as is the case in our patient, is associated with an increased risk of gastric, colorectal, hepatobiliary, breast and endometrial cancer (Win et al., 2016). There has not been an association between carrier status and ovarian carcinoma to date. The overall low tumor mutation burden, 54% *MUTYH* p.G396D variant allele fraction, and paucity of G:C to T:A transversions (single bases substitution signature 36) in our patient’s tumor suggest biallelic *MUTYH* deficiency was likely not a distinctive driver of her mucinous ovarian carcinoma. However, the role of her carrier status with regard to her overall risk of developing this tumor is uncertain.

This case raises multiple lingering questions regarding the management of patients with mucinous ovarian carcinoma, including the role of HER2-targeted therapy in the treatment of advanced stage disease, the ideal management and prognostication of patients with stage IC disease, and the genetic and environmental risk factors specific to mucinous ovarian tumors. From a precision oncology standpoint, two molecular alterations in this patient’s tumor impacted the course of her care – the finding of p53 mutant immunohistochemistry after initial resection and the *ERBB2* amplification, suggesting that evaluation for these alterations may be useful to guide clinical decision making. Proposed scoring algorithms exist for immunohistochemical assessment of these alterations with high levels of genetic concordance (Gorringe et al., 2020, McAlpine et al., 2009, Kang et al., 2021) but have not been assessed in a prospective fashion. Interestingly, increased genome wide copy number alterations including specifically 9p loss, have been implicated as markers of aggressive mucinous carcinomas (Cheasley et al., 2019) and both features were identified on the tumor’s second sequencing. As the spectrum of HER2-targeted therapies continue to expand, including novel antibody-drug conjugates such as trastuzumab deruxtecan (which showed an objective response of 36.8% in ovarian cancers with centrally confirmed ≥+2 HER2 expression by IHC Meric-Bernstam et al., 2023), it will be important to include patients with mucinous ovarian carcinoma in further tumor-agnostic studies. Ultimately, this case highlights the ongoing need for further prospective evaluation of targeted therapeutics in mucinous ovarian carcinoma with special attention paid to the identification and treatment of patients with *ERBB2*-amplified tumors.

## 4 Consent

Informed consent was obtained from the patient for discussion of her case in this report.

## Declaration of Competing Interest

The authors declare that they have no known competing financial interests or personal relationships that could have appeared to influence the work reported in this paper.
